# Exercise training and burdock root (*Arctium lappa* L.) extract independently improve abdominal obesity and sex hormones in elderly women with metabolic syndrome

**DOI:** 10.1038/s41598-021-84301-x

**Published:** 2021-03-04

**Authors:** Min-Seong Ha, Jang Soo Yook, Minchul Lee, Kazuya Suwabe, Woo-Min Jeong, Jae-Jun Kwak, Hideaki Soya

**Affiliations:** 1 Department of Sports Culture, College of the Arts, Dongguk University-Seoul, 30 Pildong-ro 1-gil, Jung-gu, Seoul, 04620 Republic of Korea; 2 Division of Sports Neuroscience, Advanced Research Initiative for Human High Performance (ARIHHP), Faculty of Health and Sport Sciences, University of Tsukuba, 1-1-1 Tennoudai, Tsukuba, Ibaraki 305-8574 Japan; 3 Laboratory of Exercise Biochemistry and Neuroendocrinology, Faculty of Health and Sports Sciences, University of Tsukuba, 1-1-1 Tennoudai, Tsukuba, Ibaraki 305-8574 Japan; 4 Center for Functional Connectomics, Brain Research Institute, Korea Institute of Science and Technology (KIST), 5 Hwarang-ro 14-gil, Seongbuk-gu, Seoul, 02792 Republic of Korea; 5 Department of Sports Medicine, College of Health Science, CHA University, 120 Haeryong-ro, Pocheon-si, Gyeonggi-do 11160 Republic of Korea; 6 WellCare Korea Co. Ltd., 26 Wadong-ro, Danwon-gu, Ansan-si, Gyeonggi-do 15265 Republic of Korea; 7 Department of National Defense Technology, Woosuk University, Daehak-ro 66, Jincheon-eup, Jincheon-gun, Chungcheongbuk-do 27841 Republic of Korea; 8 Faculty of Health and Sport Sciences, Ryutsu Keizai University, Ibaraki, 301-8555 Japan

**Keywords:** Biochemistry, Physiology, Biomarkers, Diseases, Endocrinology, Health care, Risk factors

## Abstract

The prevalence of metabolic syndrome (MS) is increasing among the elderly, and new lifestyle-based treatment strategies are warranted. We conducted a randomized, double-blind controlled trial of the effects of aquatic exercise (AE) and/or consumption of burdock root extract (BE) on body composition and serum sex hormones, i.e., testosterone, estradiol, sex hormone-binding globulin (SHBG), and dehydroepiandrosterone-sulfate (DHEA-S) in elderly women with MS. The percentage of abdominal fat was decreased in the AE group. Waist circumference was increased in the control (CON) group, but not in the other groups. SHBG and estradiol levels were enhanced by both AE and BE and correlated with changes in fat-related body composition. DHEA-S levels only increased in the BE group, which was consistent with changes in lean body mass. Testosterone levels decreased in the CON group, which correlated with changes in lean body mass, skeletal muscle mass, body fat, and waist circumference. Our findings suggested that the combined AE/BE intervention exerted no synergistic and/or additive effects on any sex-related outcome measures in elderly women with MS.

## Introduction

Metabolic syndrome (MS) is a group of metabolic disorders including hypertension, insulin resistance, impaired glucose tolerance, and abdominal obesity, and is associated with a high incidence of cardiovascular disease (CVD) and type 2 diabetes^1, 2^. The prevalence of MS increases with age, and is particularly high among the elderly^3, 4^, in which the increase in MS-associated risk factors, including central obesity and CVD, is higher in women than that in men^5, 6^.

In general, postmenopausal women exhibit considerable alterations in sex hormones, which may be related to abdominal adiposity, thus increasing the likelihood of MS. There is increasing evidence that changes in sex hormones contribute to MS pathophysiology^7^. Dehydroepiandrosterone (DHEA) and its sulfate ester (DHEA-S), the most abundant circulating steroid hormone produced by adrenal glands, are converted to testosterone and estrogen^8^. Although the precise physiological function of DHEA-S is not fully understood, its serum levels decline with age, and this decrease is associated with increased waist circumference, a diagnostic indicator of MS in elderly women^9^. Low levels of sex hormone-binding globulin (SHBG), which binds to testosterone and estradiol, are associated with high MS prevalence in postmenopausal women^10^.

Lifestyle changes commonly recommended as a first-line intervention for MS prevention and treatment include regular physical exercise and a healthy diet^11^. Several meta-analyses have shown that aerobic exercise has a positive effect on MS profiles, thereby affecting body composition, cardiorespiratory fitness, insulin resistance, as well as sex hormones^12–14^. Although there are controversial issues regarding the effects of physical exercise on sex hormone among elderly women, several studies have revealed that estradiol, testosterone, and SHBG levels are enhanced after adopting an active and healthy lifestyle^15–17^. Additionally, we observed that water-based aquatic exercise (AE), an acceptable compromise between exercise effectiveness and safety intervention for the elderly, improved fitness and vascular-related factors in elderly women without MS^18^, suggesting that regular AE might also be beneficial in elderly women with MS.

Most studies have demonstrated that appropriate combinations of exercise and diet reduce the clinical prevalence of MS^19^. However, there is a lack of scientific information regarding the effects on MS of combined interventions involving physical exercise and the consumption of specific dietary components. Among potentially beneficial dietary components, we focused on burdock (*Arctium lappa* L.), a fusiform brown root containing arctiin, luteolin, and quercetin rhamnoside, which is a remarkable source of proteins, potassium, calcium, and folate, and is rich in phytochemicals^20, 21^. As a traditional herbal medicine, burdock has been used in Asia as well as western countries for centuries. Each part of the burdock plant has a different composition, especially in terms of bioactive compounds. Indeed, various biologically active compounds, such as terpenoids (beta-eudesmol, C15H24O, present in the fruit), sterols (sitosterol-beta-d-glucopyranoside, C35H60O6, contained in the root), lignans, polyphenols, and fructans, are found in the plant complex^22^. Burdock possesses antioxidant and anti-inflammatory properties and is reportedly pharmacologically active as an anti-diabetic agent, which improves blood lipid profiles, hypoglycemia, and hyperinsulinemia^23, 24^. Particularly, one specific burdock component, arctiin, has been found to reduce body weight and adipose tissue through anti-adipogeneic effects by activating the AMP-activated protein kinase (AMPK) pathway in obese mice induced by high-fat diet^25^. These reports suggest that burdock is potentially beneficial for obesity and diabetes that is closely linked with MS.

Recently, we found that the combination of burdock root extract (BE) and AE exerted positive effects on vascular function and related hormones in elderly women without MS^18, 26^. Considering the potential health-beneficial effects of the AE and BE, the purpose of this study was to investigate the impact of AE and BE, individually or in combination, on body composition and sex-related hormone levels in elderly women with MS. We hypothesized that a 16-week combined AE/BE intervention in elderly women with MS would either additively or synergistically improve body composition, and induce beneficial changes in the levels of sex-related hormones.

## Results

### Effects of the interventions on MS criteria parameters

First, there were no significant differences in baseline age, height, and weight, between the control and the intervention groups (Table 1 and Table S1). Regarding MS features, clinical characteristics such as waist circumference, BMI, levels of triglycerides, HDL-C, glucose, and systolic blood pressure and diastolic blood pressure did not differ between groups, and were close to the upper limits established by the National Cholesterol Education Program (NCEP) Adult Treatment Panel (ATP) III criteria^27^. Thus, all subjects exhibited similar metabolic alterations.

**Table 1:** Effects of a 16-week aquatic exercise (AE) and burdock root extract (BE) intervention on metabolic syndrome and body composition parameters.

Variable	Group	Pre		Post		Effect size Cohen's *d*	Interaction	Main	
		Mean	SD	Mean	SD		*p*-value	*p*-value	
Age (years)	CON	76.71	5.91						
	AE	75.83	4.26						
	BE	73.80	5.81						
	AE + BE	74.17	4.40						
TG (mg/dl)	CON	167.14	62.04	165.57	62.09	− 0.03	0.001^††^	T	0.000^†††^
	AE	164.33	40.98	131.33	36.01**	− 0.81			
	BE	121.00	60.17	109.20	49.65	− 0.20		G	0.328
	AE + BE	179.50	22.85	119.50	18.16***	− 2.63			
HDL-C (mg/dl)	CON	54.57	10.06	53.14	7.69	− 0.14	0.026^†^	T	0.121
	AE	49.00	20.46	48.67	18.96	− 0.02			
	BE	46.20	14.13	47.36	7.64	0.08		G	0.767
	AE + BE	48.17	6.62	55.17	5.56**	1.06			
Glucose (mg/dl)	CON	111.14	25.37	120.71	29.20	0.38	0.007^††^	T	0.037^†^
	AE	123.67	24.83	96.70	14.39**	− 1.09			
	BE	95.60	14.15	95.80	4.82	0.02		G	0.190
	AE + BE	103.50	24.19	88.83	10.76	− 0.61			
Systolic BP (mmHg)	CON	140.57	24.17	147.57	13.26	0.29	0.233	T	0.141
	AE	143.83	9.99	133.33	11.06	− 1.05			
	BE	159.20	27.91	141.60	17.52	− 0.63		G	0.430
	AE + BE	161.17	22.44	150.83	30.69	− 0.46			
Diastolic BP (mmHg)	CON	73.86	7.36	76.14	8.19	0.31	0.105	T	0.038^†^
	AE	73.17	8.64	76.50	8.94	0.39			
	BE	87.60	15.92	81.20	9.04	− 0.40		G	0.057
	AE + BE	80.17	5.46	80.50	9.03	0.06			
BMI (kg/m^2^)	CON	24.78	1.06	24.76	0.94	− 0.02	0.128	T	0.267
	AE	23.94	1.54	24.02	1.50	0.05			
	BE	26.54	2.90	26.03	3.30	− 0.18		G	0.304
	AE + BE	26.17	3.41	26.22	3.43	0.02			
Waist circumference (cm)	CON	88.29	3.38	91.71	3.25*	1.01	0.003^††^	T	0.970
	AE	90.92	3.41	88.17	4.07	− 0.81			
	BE	92.30	5.60	94.20	7.36	0.34		G	0.679
	AE + BE	90.25	9.87	87.77	9.74	− 0.25			
% body fat	CON	35.32	3.07	38.43	7.62	1.01	0.161	T	0.390
	AE	31.66	6.58	28.89	6.62	− 0.42			
	BE	36.90	3.76	35.50	3.73	− 0.37		G	0.041^†^
	AE + BE	39.42	4.46	36.76	4.42	− 0.60			
% abdominal fat	CON	0.88	0.03	0.92	0.04	1.33	0.005^††^	T	0.486
	AE	0.87	0.06	0.81	0.10	− 1.00*			
	BE	0.92	0.09	0.94	0.08	0.22		G	0.088
	AE + BE	0.90	0.03	0.89	0.03	− 0.33			
Lean body mass (kg)	CON	37.41	2.17	35.51	3.77	− 0.88	0.112	T	0.560
	AE	39.13	3.82	40.77	2.45	0.43			
	BE	40.62	5.91	40.62	5.68	0.00		G	0.151
	AE + BE	36.70	2.72	38.37	2.56	0.61			

Repeated measures ANOVA (time × group) showed a main effect and interaction on all parameters.

*CON* control, *BMI* body mass index, *TG* triglycerides, *HDL-C* high density lipoprotein cholesterol, *BP* blood pressure, *T* time effect, *G* group effect.

Values are the mean ± SD. ^†^*p* < 0.05, ^††^*p* < 0.01, ^†††^*p* < 0.001. *p < 0.05, **p < 0.01, ***p < 0.001 vs. pre-trial. Effect size range: |0.20| ≦ small < |0.50| < medium < |0.80| ≦ large^56^.

Changes in MS criteria parameters combination interventions are shown in Table 1. A significantly remarkable effect of time was observed for triglycerides (*F* = 33.003, *p* = 0.000) and glucose (*F* = 4.987, *p* = 0.037). A significant time-group interaction was identified for waist circumference (*F* = 6.435, *p* = 0.003), triglycerides (*F* = 8.162, *p* = 0.001), HDL-C (*F* = 3.795, *p* = 0.026), and glucose (*F* = 5.446, *p* = 0.007). Post-hoc analysis revealed that control (CON) women exhibited a significant increase in waist circumference (*t* = 2.950, *p* = 0.031), while a significant decrease in triglyceride levels was observed in AE and AE/BE women (*t* = 3.590, *p* = 0.007; *t* = 6.527, *p* = 0.000). Additionally, AE/BE women showed a significant increase in HDL-C (*t* = 3.572, *p* = 0.008), and AE women a significant decrease in glucose levels (*t* = 3.807, *p* = 0.004).

### Effects of the interventions on body composition

**Figure 1:**
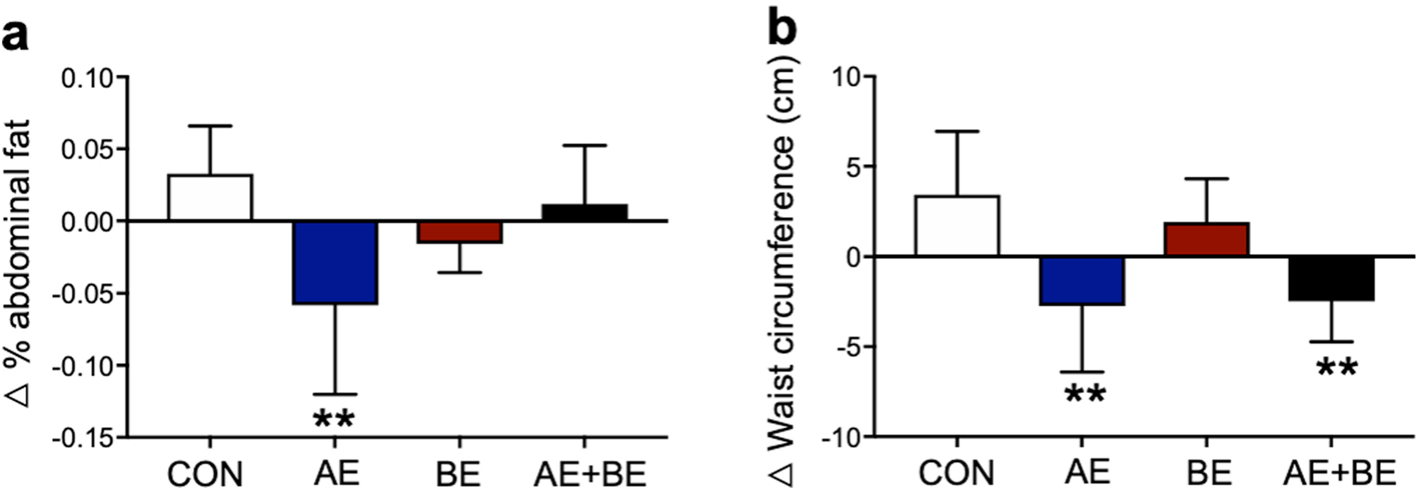
Changes in abdominal fat percentage and waist circumference. (**a**) Significant differences were observed between aquatic exercise (AE) and control (CON) subjects in the delta (Δ) values of % abdominal fat (^**^p < 0.01). (**b**) Significant differences were observed between AE + BE (burdock root extract) and CON subjects in the delta (Δ) value of waist circumference (^**^p < 0.01). Data are expressed as mean ± SD.

Recently, body composition parameters were found to be closely associated with MS risk^28^. First, we measured BMI, body fat mass percentage, lean body mass, skeletal muscle mass, abdominal fat (%), and waist circumference before and after the 16-week interventions. The percentage of abdominal fat and waist circumference exhibited a significant time-group interaction (*F* = 5.950, *p* = 0.005; *F* = 6.435, *p* = 0.003) (Table 1). A significantly remarkable effect of group was observed for the percentage of body fat (*F* = 3.315, *p* = 0.041). Post-hoc analysis showed that AE significantly decreased abdominal fat percentage (*t* = 3.395, *p* = 0.011) with a large effect size (*d* =  − 1.00). After the intervention period, we observed increase in waist circumference values in the CON group with a large effect size (*d* = 1.01), but not in the AE, BE, or AE + BE subjects. Furthermore, beneficial changes (Δ) in body composition parameters were observed. The participants undergoing AE demonstrated a significantly greater reduction in the percentage of abdominal fat in comparison to CON subjects (*t* = 3.894, *p* = 0.002; Fig. 1a). Moreover, subjects exposed to the AE alone or the AE/BE combination showed a significant decrease in waist circumference, compared to the CON group (*t* = 3.612, *p* = 0.005; *t* = 3.456, *p* = 0.007, respectively; Fig. 1b). Thus, our result suggest that the intervention of AE and/or BE have positive effects in altering MS risks, particularly in reducing abdominal obesity factors.

### Effects of the interventions on sex hormones

**Figure 2:**
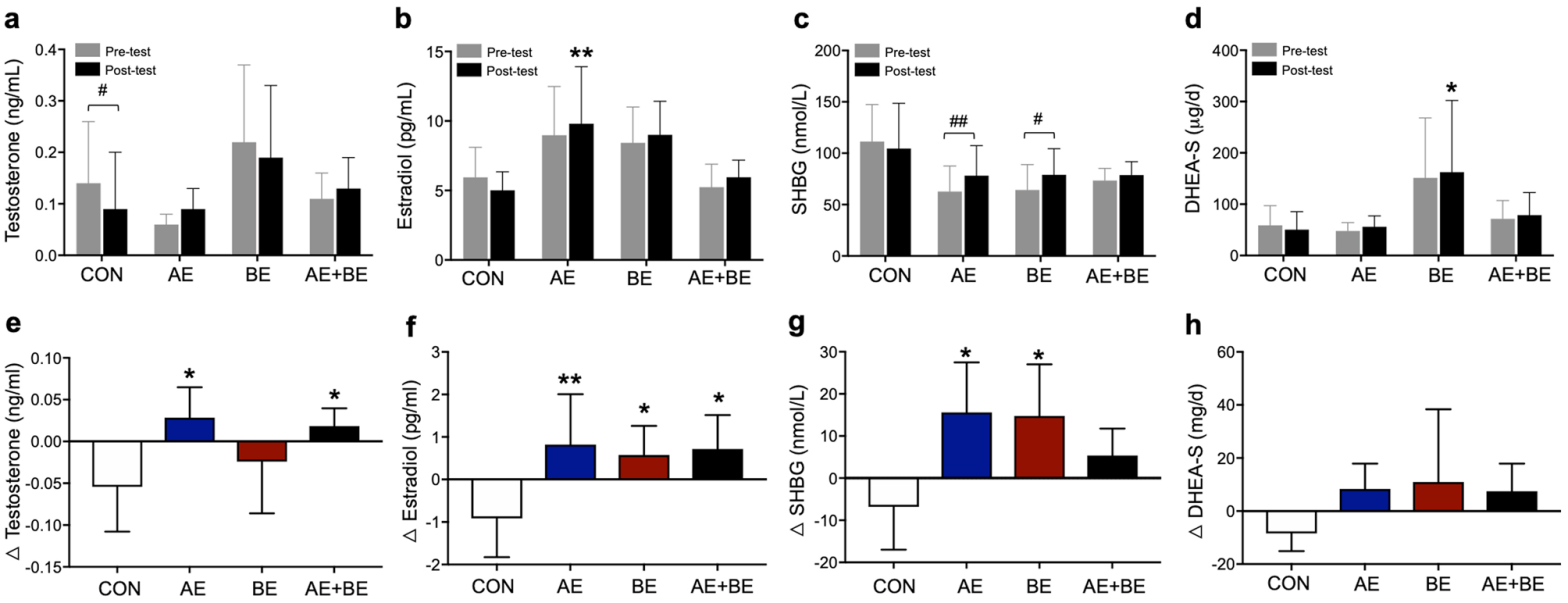
Effects of 16-week aquatic exercise (AE) and burdock root extract (BE) interventions on serum sex hormones. (**a**) Testosterone levels in the control (CON) group were decreased after the 16-week intervention period (^#^*p* < 0.05). (**b**) The post-test levels of estradiol were higher in AE (^**^p < 0.01) compared to the CON group. (**c**) Sex hormone-binding globulin (SHBG) levels were increased in both AE (^##^*p* < 0.01) and BE subjects (^#^*p* < 0.05), but not in the AE + BE group. (**d**) The post-test levels of dehydroepiandrosterone sulfate (DHEA-S) were higher in the BE than in the CON group (*p < 0.05). (**e**) A significant increase in the delta (Δ) value of testosterone was observed in the AE and AE + BE groups compared to the CON group (^*^p < 0.05). (**f**) A significant increase in the delta (Δ) value of estradiol was detected in AE (^**^p < 0.01), BE (^*^p < 0.05), and AE + BE women (^*^p < 0.05) compared to CON women. (**g**) A significant increase in the delta (Δ) value of SHBG was observed in the AE and BE groups compared to the CON group (^*^p < 0.05). (**h**) No significant changes in DHEA-S levels were observed between the groups. Data are presented as mean ± SD.

Previous reports have suggested that altered levels of endogenous sex hormones are risk factors for MS^7^. We determined the serum levels of testosterone, estradiol, SHBG, and DHEA-S to investigate the effects of AE and BE interventions on sex hormones. The results are presented in Fig. 2 as a list. A significantly remarkable effect of group for estradiol (*F* = 4.148, *p* = 0.019; Fig. 2b) and DHEA-S (*F* = 3.145, *p* = 0.048; Fig. 2d), as well as a significantly remarkable effect of time for SHBG (*F* = 11.607, *p* = 0.003; Fig. 2c) were observed. Significant time-group interactions were identified for testosterone (*F* = 4.475, *p* = 0.15; Fig. 2a), estradiol (*F* = 5.213, *p* = 0.008; Fig. 2b), and SHBG (*F* = 6.517, *p* = 0.003; Fig. 2c). Post-hoc analysis showed that only CON women exhibited a significant decrease in testosterone levels after the intervention period (*t* = 3.124, *p* = 0.021; Fig. 2a), while the AE group showed a large effect size (*d* = 1.26), but no statistically significant differences. Post-test estradiol levels were higher in the AE (*t* = 3.407, *p* = 0.009) compared to the CON group (Fig. 2b). Post-hoc analyses revealed a significant increase in SHBG levels in AE (*t* = 3.698, *p* = 0.005) and BE women (*t* = 3.198, *p* = 0.018) after the test (Fig. 2c). A significant group effect was observed for post-test serum levels of DHEA-S, as they were higher in BE compared to CON women (*t* = 2.297, *p* = 0.015; Fig. 2d). Differences (Δ) between pre and post-exercise levels of sex hormones are presented in Fig. 2. Briefly, Δ-testosterone was found to be higher in both AE (*t* = 3.262, *p* = 0.01) and AE + BE (*t* = 2.867, *p* = 0.025) compared to CON women (Fig. 2e), and Δ-estradiol was higher in AE (*t* = 3.385, *p* = 0.008), BE (*t* = 2.762, *p* = 0.031), and AE + BE group (*t* = 3.117, *p* = 0.012; Fig. 2f). AE (*t* = 3.9, *p* = 0.002) and BE subjects (*t* = 3.569, *p* = 0.011) exhibited a significantly greater Δ-SHBG compared to the CON group (Fig. 2g). However, Δ-DHEA-S showed no significant changes between the groups (Fig. 2h).

### Correlations between body compositon and sex hormones

**Figure 3:**
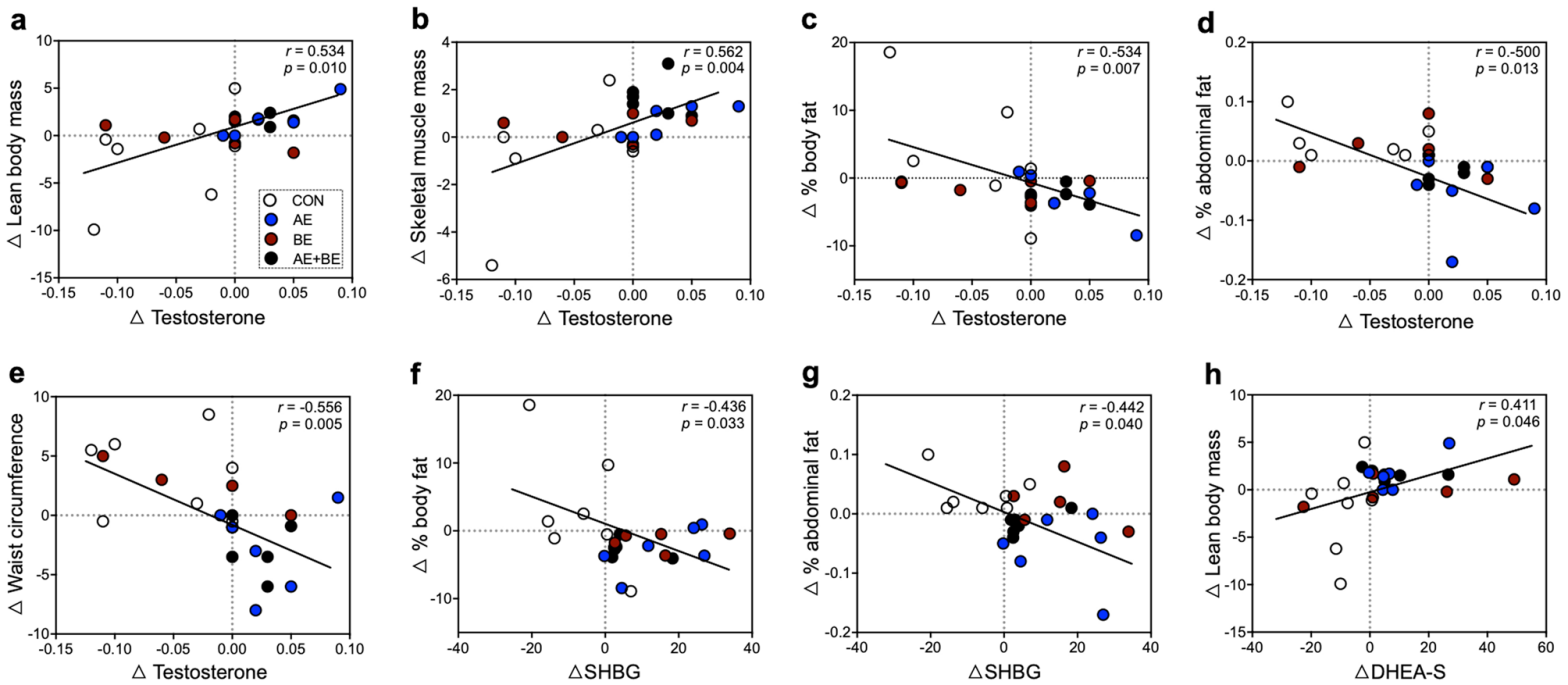
Correlation between body composition indices and sex hormones. (**a**–**e**) Δ-testosterone was correlated with Δ-lean body mass, Δ-skeletal muscle mass, Δ-percentage of body fat, Δ-percentage of abdominal fat and, Δ-waist circumference. (**f**, **g**) Δ-SHBG (sex hormone-binding globulin) was correlated with Δ-percentage of body fat and Δ-percentage of abdominal fat. (**h**) A significant correlation was observed between Δ-DHEA-S (dehydroepiandrosterone sulfate) and Δ-lean body mass. Correlation analysis was performed (Pearson’s correlation coefficients). Each circle represents an individual subject (*n* = 24).

To determine whether the levels of specific sex hormones were related to body composition parameters in response to the 16-week intervention, we analyzed the correlation between the Δ values of body composition variables and individual sex hormones, respectively (Table S2 and Fig. 3). Δ-testosterone was positively correlated with Δ-lean body mass (*r* = 0.534, *p* = 0.010; Fig. 3a) and Δ-skeletal muscle mass (*r* = 0.562, *p* = 0.004; Fig. 3b), and negatively correlated with Δ-% body fat (*r* = − 0.534, *p* = 0.007; Fig. 3c), Δ-% abdominal fat (*r* = − 0.500, *p* = 0.013; Fig. 3d), and Δ-waist circumference (*r* = − 0.556, *p* = 0.005; Fig. 3e). However, Δ-estradiol did not correlate with body composition parameters (Table S2). Moreover, Δ-SHBG was negatively correlated with Δ-% body fat (*r* = − 0.436, *p* = 0.033; Fig. 3f) and Δ-% abdominal fat (*r* = − 0.422, *p* = 0.040; Fig. 3g). Finally, a significant relationship was observed between Δ-DHEA-S and Δ-lean body mass (*r* = 0.411, *p* = 0.046; Fig. 3h).

## Discussion

Exercise regimens reportedly exert a significant effect on MS indicators^29^, and are considered as a valuable non-pharmacological approach to MS management. The aim of this study was to investigate the combined effects of AE and BE on body composition and serum gonadal steroid hormones, as well as their correlation in elderly women with MS. Based on our previous findings^18, 26^, we hypothesized that the combined effects of AE and BE would lead to additive or synergistic improvement in MS-related body composition and sex hormone levels in elderly women with MS. The results indicated that both AE and BE interventions individually decreased abdominal fat and waist circumference, which induced beneficial changes in the levels of testosterone, estradiol, and SHBG. However, we found no additive or synergistic effect on any of the MS-related outcome measures. Regardless of the contribution of a ceiling effect to these results, we found that AE or BE had potential as non-pharmacological interventions for improving MS symptoms in elderly women.

Abdominal obesity is associated with MS, and is currently the main clinical criterion for the assessment of individual MS risk according to the NCEP-ATP III guidelines^30^. Waist circumference is widely used as a surrogate marker of abdominal obesity in the elderly, including the elderly constituting the population in Asia^31^. In this study, the CON group showed a substantial increase in waist circumference after the 16-week intervention period, and both AE and the AE/BE combination resulted in a significant reduction of Δ-waist circumference (Fig. 1b). Notably, a 16-week aqua aerobic exercise regimen has previously been shown to reduce waist circumference in subjects with similar characteristics to our cohort^32^. However, we found that AE alone, but not BE alone, significantly reduced the percentage of abdominal fat, with a large effect size (Table 1). Therefore, AE may be more effective compared to both BE and the AE/BE combination at reducing MS-related abdominal obesity.

Many studies have reported associations between the levels of serum sex hormones and the risk of MS^7, 33^. Weinberg et al.^10^ showed that postmenopausal women with MS exhibited higher blood levels of testosterone (0.2 ng/mL) and estradiol (9.2 ng/mL), consistent with the levels of sex hormones detected in our study. Moreover, in the latter study, acute exercise was found to further increase the levels of these hormones^15^, while chronic exercise did not affect the levels of these hormones^34^. We found that in elderly women with MS, the change in testosterone from pre- to post-exercise was significantly greater in the AE and AE + BE groups compared to the CON group (Fig. 2e). All intervention groups showed greater Δ-values for estradiol compared to the CON group. However, the AE/BE combination did not produce any synergistic effect on the level of Δ estradiol (Fig. 2f). In addition to the correlation between body composition parameters and sex hormones, Δ-testosterone significantly correlated with the delta values for waist circumstance, percentage of abdominal fat, percentage of body fat, skeletal muscle mass, and lean body mass (Table S2 and Fig. 3), suggesting that the beneficial effects of AE on abdominal obesity were mainly mediated by changes in testosterone metabolism.

Consistent with its role as a testosterone transporter, SHBG has been associated with the risk of MS. Indeed, low levels of serum SHBG were observed in elderly men and women with MS^10, 35^. Recently, cross-sectional studies showed that serum levels of SHBG were inversely associated with waist circumference and waist-to-height ratios in Korean and Chinese individuals, respectively^36^. Thus, since SHBG concentration is a putative biomarker of abdominal obesity-related MS, we investigated the impact of AE and BE interventions on serum SHBG levels. Both interventions separately increased SHBG levels, but their combination did not produce synergistic effects (Fig. 2c). Additionally, SHBG exhibited a higher delta value in the AE and BE groups compared to the CON group (Fig. 2g), which was significantly correlated with Δ-percentage of abdominal fat and Δ-percentage of body fat (Fig. 3g). Recent in vitro experiments in adipocytes and macrophage cells have shown that SHBG protects against inflammation and lipid accumulation^37^. Further, recent in vivo studies using human SHBG transgenic mice crossed with type-2 diabetic mice (C57BL/ksJ-db/db) have shown that hepatocyte nuclear factor 4 alpha (HNF-4α) and peroxisome proliferator-activated receptor gamma (PPARα) in liver are involved in obesity progression, while human SHBG overexpression partly prevents the increase in body and liver weight, as well as in the proportion of adipose tissue^38, 39^. Our findings support the notion that AE and BE, by increasing endogenous circulating SHBG, are potential non-pharmacological options against obesity and MS-related fatty liver disease.

A considerable number of studies have documented that low levels of the adrenal steroids, DHEA, and DHEA-S, are associated with risk factors for MS, such as insulin resistance and obesity^35, 40, 41^. In contrast, DHEA administration led to beneficial effects in obesity-related MS parameters in elderly women^42^. In line with these studies, we observed alterations in the serum levels of DHEA-S in response to AE and BE interventions. However, these changes were relatively small, and only BE resulted in a clear elevation of DHEA-S levels compared to control subjects. Although we did not detect any significant change in DHEA-S due to the intervention, DHEA-S showed a positive correlation with Δ-lean body mass (Fig. 3h). Considering that DHEA can be converted to testosterone and estradiol^43^, our findings suggest that changes in the circulating levels of these hormones may contribute to the beneficial effects of AE and BE on abdominal obesity-related MS.

MS is generally caused by a number of pathophysiological mechanisms combined with lifestyle-related factors, such as unhealthy dietary patterns and lack of physical activity^44^. Among the consequences of the aforementioned lifestyle changes, abdominal obesity is the most predominant causative factor^45^. Moreover, sex hormone imbalance in the adipose tissue is involved in MS pathophysiology^35^. We therefore analyzed several parameters to assess the effects of combined BE and AE intervention on the status of abdominal obesity. The percentage of abdominal fat was decreased after AE intervention and waist circumference increased in CON group, but not in the intervention groups (Table 1), suggesting that AE and/or BE had positive effects in inhibiting an increase of MS-related risk factors, particularly abdominal obesity.

A number of studies have investigated the effects of dietary supplements on sex hormones related to conditions of age as well as noncommunicable diseases, because nutrients have important roles for hormone metabolism in the endocrine system^46^. Furthermore, sex-related endogenous hormones are associated with adiposity in postmenopausal women with diabetes^47^. A clinical study showed that administration of DHEA had beneficial effects on abdominal fat and insulin sensitivity in elderly subjects with MS^48^. Supplementation with nutrients containing vitamin E, selenium, vitamin C, and coenzyme Q10 showed no overall significant effects on sex-related hormones^49^, and vitamin E supplementation alone in elderly subjects did not affect serum levels of DHEA-S^50^. However, we observed that BE intervention significantly increased DHEA-S levels (Fig. 2d). Although it is uncertain what nutritional component from BE induces the increase in DHEA-S levels, BE supplementation may have similar effects as DHEA replacement therapy, in addition to reducing insulin resistance.

Although our study provides novel lifestyle-based insights into MS management in elderly women, the following limitations must be acknowledged. First, the small sample size does not allow for generalization. However, this study may be an important conceptual basis to explore the combined effects of AE and BE on elderly women with MS in future, larger experiments. Second, we did not investigate dose–response BE effects. Therefore, additional positive effects of BE may be expected from the application of higher BE doses. Further studies are warranted to address these issues.

## Conclusions

In summary, this study is the first to investigate the effects of a combination of AE plus the natural dietary component, BE, on body composition and sex hormones in elderly women with MS. Both AE and BE independently improved abdominal fat and waist circumference, and altered the serum levels of sex hormones, such as testosterone and estradiol, which in turn are known to be implicated in central obesity-related MS in elderly women. However, contrary to our hypothesis, the combination of AE and BE did not produce any additive or synergistic effect on the investigated parameters.

## Materials and methods

### Study participants

Thirty-two elderly women volunteers with MS from South Korea were initially enrolled (average age: 74.31 ± 5.2). Twenty-four subjects (average age: 75.25 ± 4.96) were finally selected for the 16-week experiment and categorized as follows: (1) control group (CON: *n* = 7), (2) aquatic exercise group (AE: *n* = 6), (3) burdock root extract ingestion group (BE: *n* = 5), and (4) combination of aquatic exercise and burdock root extract ingestion group (AE + BE: *n* = 6). The baseline characteristics of study participants are shown in Table 1. Before commencement of the study, the participants were informed of the research purpose and intentions, and informed consent forms were obtained from all participants and approved by the National Bioethics Committee of the Pusan National University (PNU IRB/2015_22), in accordance with the Declaration of Helsinki Declaration and the 2010 Consolidated Standards of Reporting Trials statement^51^. This trial was retrospectively registered in the University Hospital Medical Information Network Clinical Trial Registry (Japan, registration 15/04/2020 UMIN000040170).

### Study design

A 16-week intervention study comprising four randomized, double-blind controlled trials investigating the effect of dietary BE supplementation, with or without exercise intervention, was conducted. Pre- and post-tests were performed at the same time each day to minimize temperature-related changes. MS diagnosis was based on the NCEP-ATP III guidelines, recommending the presence of at least three of the following six criteria^28^. After baseline measurements, all participants were randomly divided into four groups. The following parameters were tested before and after the 16-week intervention: body composition (weight, BMI, % body fat, fat body mass, lean body mass, skeletal muscle mass, % abdominal fat, and waist circumference), and circulating hormones (DHEA-S, SHBG, testosterone, estradiol). For a comprehensive evaluation, the lifestyle habits of the subjects were also monitored by the researchers, and in particular, the control group was encouraged to maintain their usual lifestyle.

### Aquatic exercise protocol

We previously reported that aquatic exercise enhanced fitness factors and vascular function in older adults^18, 26^. Thus, we used the same protocol to the current study. The aquatic exercise program was based on recommendations of the American College of Sports Medicine^52^, and was scheduled considering the age of subjects. Exercise was performed three times per week for 16-weeks, following a 1–6-week adjustment period at a swimming pool. The program consisted of a 5-min warm-up and a 5-min cool-down exercise session, and a 40-min main exercise session with individualized loads corresponding to 30–40% heart rate reserve (HRR) at a rating of perceived exertion (RPE) of 9–10 for weeks 1–5, 40–50% HRR (RPE 11–12) for weeks 6–10, and 50–60% HRR (RPE 13–14) for weeks 11–16. Heart rates were monitored using a heart rate monitor watch (Polar RS400sd; model APAC, 90026360; Polar, NY, USA) and Borg’s RPE^53^ was checked twice during the exercise session.

### Burdock root extract sampling and ingestion

BE samples were prepared based on methods optimized and described in a previous study^18, 26^. After the addition of 4 kg of fresh burdock root harvested in the Sancheong region (Gyeongnam, South Korea) and 6000 mL of water to an extractor, extraction was performed for 3 h at 100 °C at a pressure of 0.7 kg/cm^2^. The main ingredients of BE were water (98.02% ± 0.02%), crude ash (0.10% ± 0.00%), crude fat (1.12% ± 0.00%), crude protein (0.20% ± 0.00%), crude fiber (0.03%), calcium (0.004% ± 0.00%), and phosphorus (0.009% ± 0.00%) (Pukyong National University Feed & Foods Nutrition Research Center, Busan, South Korea). BE administration schedule was based on the advice of an oriental medical doctor. Specifically, the participants consumed one 100-mL dose of BE 3 times a day after each meal (breakfast, lunch, and dinner), for a total of 300 mL of BE per day for 16 weeks.

### Body composition and blood biochemical analysis

Participants were advised to refrain from eating after 8:00 p.m. on the day before the test, and the test was performed between 8:00 and 9:00 a.m. according to the procedures recommended by the American College of Sports Medicine^54^. Bioelectrical impedance, measured with the Inbody 720 device (Biospace, Seoul, Korea), was used to assess body composition. The study participants were instructed to assume a comfortable standing position with their feet slightly apart on the instrument while wearing casual clothing; all metal objects were removed. Blood samples were collected using EDTA tubes and needles at two time points, i.e., before and after the 16-week intervention. After collecting 10 mL of blood from an antebrachial vein, the serum was isolated for analysis of sex hormones.

### Statistical analysis

The required sample size was calculated using the G-power version 3.1 Windows program (Kiel University, Kiel, Germany), based on a 0.25-point effect size (default), an alpha level of 0.05, and 40% power^55^. The results indicated that 20 participants were requisite for the study; assuming a dropout rate of 25%, the sample size was set to 32 participants. All data were expressed as mean ± standard deviation (SD). Two-way repeated ANOVA was performed to evaluate the differences between groups and time for absolute value of body composition and sex hormones, followed by Bonferroni’s multiple comparison tests for post-hoc analysis. One-way ANOVA with Dunnett’s multiple comparison tests was used to analyze the delta (Δ) change. Correlations between body composition and sex hormones were calculated by Pearson’s correlation analysis. A *p* < 0.05 was considered statistically significant. Effect sizes (Cohen's *d*) between pre- and post-test data were expressed as mean changes^56^.

### Ethical approval

All procedures and protocols performed in studies involving human participants were by the ethical standards of the institutional and/or national research committee and with the 1964 Helsinki Declaration and the 2010 Consolidated Standards of Reporting Trials statement^46^ were approved by the National Bioethics Committee of Pusan National University (PNU IRB/2015_22). This trial was retrospectively registered in the University Hospital Medical Information Network Clinical Trial Registry (Japan, registration 15/04/2020 UMIN000040170).

## Supplementary Information

Supplementary Information.

## Data Availability

Data and publication materials are available upon request.

## Abbreviations

MS: Metabolic syndrome
AE: Aquatic exercise
BE: Burdock root extract
DHEA-S: Dehydroepiandrosterone sulfate
SHBG: Sex hormone-binding globulin
