# Supplementary material for: Exercise training and burdock root (Arctium lappa L.) extract independently improve abdominal obesity and sex hormones in elderly women with metabolic syndrome
Source: Sci Rep. 2021 Mar 4;11:5175. doi: 10.1038/s41598-021-84301-x (PMC7933410; doi:10.1038/s41598-021-84301-x)
Supplement: Supplementary file 1 — Supplementary Information. [file 41598_2021_84301_MOESM1_ESM.docx]

Exercise training and burdock root (*Arctium lappa L*.) extract independently improve abdominal obesity and sex hormones in elderly women with metabolic syndrome

Min-Seong Ha ^1,2,3,$,*^, Jang Soo Yook ^4,$^, Min Chul Lee ^5^, Kazuya Suwabe ^2,3^, Woo-Min Jeong ^6^, Jae-Jun Kwak ^7^, and Hideaki Soya ^2,3,*^

^1^ Department of Sports Culture, College of the Arts, Dongguk University-Seoul, 30 Pildong-ro 1-gil, Jung-gu, Seoul 04620, Republic of Korea; haminseong@dgu.ac.kr (M-S.H.)

^2^ Division of Sports Neuroscience, Advanced Research Initiative for Human High Performance (ARIHHP), Faculty of Health and Sport Sciences, University of Tsukuba, 1-1-1 Tennoudai, Tsukuba, Ibaraki 305-8574, Japan; ha.minseong.fn@u.tsukuba.ac.jp (M-S.H.); suwabe.kazuya.kb@u.tsukuba.ac.jp (K.S.); soya.hideaki.gt@u.tsukuba.ac.jp (H.S.)

^3^ Laboratory of Exercise Biochemistry and Neuroendocrinology, Faculty of Health and Sports Sciences, University of Tsukuba, 1-1-1 Tennoudai, Tsukuba, Ibaraki 305-8574, Japan

^4^ Center for Functional Connectomics, Brain Research Institute, Korea Institute of Science and Technology (KIST), 5 Hwarang-ro 14-gil, Seongbuk-gu, Seoul 02792, Republic of Korea; soulyook84@gmail.com (JS.Y.)

^5^ Department of Sports Medicine, College of Health Science, CHA University, 120 Haeryong-ro, Pocheon-si, Gyeonggi-do 11160, Republic of Korea; mclee@cha.ac.kr (MC.L.)

^6^ WellCare Korea Co. Ltd., 26 Wadong-ro, Danwon-gu, Ansan-si, Gyeonggi-do 15265, Republic of Korea; woominj79@hanmail.net (W-M.J.)

^7^ Department of National Defense Technology, Woosuk University, Daehak-ro 66, Jincheon-eup, Jincheon-gun, Chungcheongbuk-do 27841, Republic of Korea; jjghsh625@gmail.com (J-J.K.)

**^$^** M-S. Ha and JS. Yook contributed equally to this work.

^*^ Correspondence: haminseong@dgu.ac.kr; Tel: +82-2-2290-1926 (M-S.H.) and soya.hideaki.gt@u.tsukuba.ac.jp; Tel.: +81-29-853-2620 (H.S.)

Supplemental material content:

**Detailed Materials and Methods**

**Supplemental Table 1**

**Supplemental Table 2**

Detailed Materials and Methods

Study participants

Thirty-two elderly women volunteers with MS from South Korea were initially enrolled (average age: 74.31 ± 5.2). The participants underwent physical examination, as well as a medical interview by a doctor to confirm that they did not practice regular physical activity, and had no history of hormone replacement therapy, drug or alcohol abuse, smoking, allergies, chronic diseases, thyroid disease, eating disorders, consumption of any supplement, or atherosclerotic cardiovascular disease. Twenty-four subjects (average age: 75.25 ± 4.96) were finally selected for the 16-week experiment: (1) control group (CON: *n* = 7), (2) aquatic exercise group (AE: *n* = 6), (3) burdock root extract ingestion group (BE: *n* = 5), and (4) combination of aquatic exercise and burdock root extract ingestion group (AE+BE: *n* = 6). The baseline characteristics of study participants are shown in Table 1. Before starting the study, the participants were fully informed of the research purpose and intentions, and consent forms were obtained from all participants and collected by the Institutional Human Research Committee of the Pusan National University (PNU IRB/2015_22), in accordance with the Declaration of Helsinki Declaration and the 2010 Consolidated Standards of Reporting Trials statement^49^. This trial was retrospectively registered in the University Hospital Medical Information Network Clinical Trial Registry (Japan, registration 15/04/2020 UMIN000040170).

Study Design

A 16-week intervention study was conducted and comprised of four randomized, double-blind controlled trial investigating the effect of dietary BE supplementation, with or without exercise intervention. Pre- and post-tests were performed at the same time each day to minimize temperature-related changes. MS diagnosis was based on the NCEP-ATP III guidelines, recommending the presence of at least three of the following six criteria^28^: waist circumference > 80 cm; serum triglycerides ≥ 150 mg/dl; serum high-density lipoprotein cholesterol (HDL-C) level ≥ 50 mg/dl; blood pressure ≥ 130/85 mmHg; plasma glucose level ≥ 110 mg/dl or BMI ≥ 25 kg/m^2^. After baseline measurements, all participants were randomly divided into four groups. The following parameters were tested before and after the 16-week intervention: body composition (weight, kg; BMI, kg/m^2^; % body fat, %; fat body mass, kg; lean body mass, kg; skeletal muscle mass, kg; % abdominal fat, %; waist circumference, cm) and circulating hormones (DHEA-S, µg/dl; SHBG, nmol/L; testosterone, ng/ml; estradiol, pg/ml). For completeness, the lifestyle habits of the subjects were also monitored by the researchers, and in particular, the control group was encouraged to maintain their usual lifestyle.

Aquatic Exercise Protocol

We previously reported that aquatic exercise enhances fitness factors and vascular function in old adults^18,26^. Thus, we applied the same protocol to the current study. The aquatic exercise program was based on recommendations of the American College of Sports Medicine

^50^, and scheduled taking into account the age of subjects. In particular, exercise was performed three times per week for 16-week, following a 1–6-week adjustment period at a swimming pool (room temperature, 30–33 °C; humidity, 70–75%; water temperature, 26-28 °C; water depth, 1.2 m). The program consisted of a 5-min warm-up and a 5-min cool-down exercise session, followed by a 40-min main exercise session with individualized loads corresponding to 30–40% heart rate reserve (HRR) at a rating of perceived exertion (RPE) of 9–10 for weeks 1–5, 40–50% HRR (RPE 11–12) for weeks 6–10, and 50–60% HRR (RPE 13–14) for weeks 11–16. The heart rate was monitored using a heart rate monitor watch (Polar RS400sd; model APAC, 90026360; Polar, NY, USA) and Borg’s RPE^53^ was checked twice during the exercise session.

Burdock Root Extract Sampling and Ingestion

BE samples were prepared based on methods optimized in a previous study^18,26^. After the addition of 4 kg of fresh burdock root harvested in the Sancheong region (Gyeongnam, South Korea) and 6,000 mL of water to an extractor, extraction was performed for 3 h at 100 °C at a pressure of 0.7 kg/cm^2^. The BE was sealed in plastic bags in 100-mL portions, and stored in a refrigerator. The main ingredients of BE were water (98.02 ± 0.02%), crude ash (0.10 ± 0.00%), crude fat (1.12 ± 0.00%), crude protein (0.20 ± 0.00%), crude fiber (0.03%), calcium (0.004 ± 0.00%), and phosphorus (0.009 ± 0.00%) (Pukyong National University Feed & Foods Nutrition Research Center, Busan, South Korea). BE administration schedule was based on the advice of an oriental medical doctor. Specifically, the participants consumed one 100-mL dose of BE, 3 times a day, after each meal (breakfast, lunch, and dinner), for a total of 300 mL of BE per day for 16-week.

Body Composition and Blood Biochemical Analysis

Body composition and blood biochemistry were analyzed by previously reported methods^24^. Participants were advised to refrain from eating after 8:00 PM on the day before the test, and the test was performed between 8:00 and 9:00 AM according to the procedures recommended by the American College of Sports Medicine^54^. Bioelectrical impedance, measured with an Inbody 720 device (Biospace, Seoul, Korea), was used to assess the body composition. The study participants were instructed to assume a comfortable standing position with their feet slightly apart on the instrument, while wearing casual clothing; all metal objects were removed. Blood samples were collected using EDTA tubes and needles at two time points: before and after the 16-week intervention. After collecting 10 mL of blood from an antebrachial vein, the blood was centrifuged for 10 min at 3,000 rpm using a Combi-514R centrifuge (Hanil, Gimpo, Korea), and the serum was isolated for analysis. The supernatant was transferred to a 1.5-ml tube and the levels of sex hormones were analyzed of sex hormones.

Statistical Analysis

The required sample size was calculated using the G-power version 3.1 Windows program (Kiel University, Kiel, Germany), based on a 0.25-point effect size (default), an alpha level of 0.05, and 40% power^55^. The results indicated that 20 participants were needed; assuming a dropout rate of 25%, the sample size was set to 32 participants.

All data were expressed as mean ± standard deviation (SD). Two-way repeated ANOVA was performed to evaluate the differences between groups and time for absolute value of body composition and sex hormones, followed by Bonferroni’s multiple comparison tests for post-hoc analysis. One-way ANOVA with Dunnett’s multiple comparison tests was used to analyze the delta (Δ) change. Correlations between body composition and sex hormones were calculated by Pearson’s correlation analysis. A *p* < 0.05 was considered statistically significant. Effect sizes (Cohen's *d*) between pre- and post-test data were expressed as mean changes. Standard interpretations of the effect size were used (*d*: |0.20|≦small<|0.50|<medium<|0.80|≦large)^56^.

**Table S1.** Effects of 16-week aquatic exercise (AE) and burdock root extract (BE) interventions on body composition.

| **Variable** | **Group** | **Pre** | | **Post** | | **Effect size  Cohen's *d*** | **Interaction** | **Main** | |
| --- | --- | --- | --- | --- | --- | --- | --- | --- | --- |
|  |  | **Mean** | **SD** | **Mean** | **SD** |  | ***p*-value** | ***p*-value** | |
| Height (cm) | CON | 152.86 | 4.41 | 152.86 | 4.41 | 0.00 | - | T | - |
|  | AE | 154.83 | 4.49 | 154.83 | 4.49 | 0.00 |  |  |  |
|  | BE | 155.60 | 5.86 | 155.60 | 5.86 | 0.00 |  | G | - |
|  | AE+BE | 152.67 | 3.20 | 152.67 | 3.20 | 0.00 |  |  |  |
| Weight (kg) | CON | 57.84 | 1.85 | 57.80 | 2.07 | -0.02 | 0.126 | T | 0.280 |
|  | AE | 57.47 | 5.45 | 57.67 | 5.60 | 0.04 |  |  |  |
|  | BE | 64.66 | 11.28 | 63.44 | 12.06 | -0.11 |  | G | 0.401 |
|  | AE+BE | 60.95 | 7.64 | 61.07 | 7.73 | 0.02 |  |  |  |
| Body fat mass (kg) | CON | 20.43 | 1.89 | 22.29 | 4.82 | 0.99 | 0.170 | T | 0.367 |
|  | AE | 18.33 | 4.66 | 16.90 | 5.03 | -0.31 |  |  |  |
|  | BE | 24.04 | 6.03 | 22.82 | 6.63 | -0.20 |  | G | 0.172 |
|  | AE+BE | 24.25 | 5.85 | 22.70 | 5.75 | -0.27 |  |  |  |
| Skeletal muscle mass (kg) | CON | 19.97 | 1.35 | 19.31 | 1.54 | -0.49 | 0.057 | T | 0.094 |
|  | AE | 22.07 | 1.75 | 22.70 | 1.64 | 0.36 |  |  |  |
|  | BE | 21.90 | 3.54 | 22.30 | 3.36 | 0.11 |  | G | 0.073 |
|  | AE+BE | 19.52 | 1.50 | 21.18 | 2.12 | 1.11 |  |  |  |

Repeated measures ANOVA (time × group) showed a main effect and interaction on body composition. Values are the mean ± SD. Effect size range: |0.20|≦small<|0.50|<medium<|0.80|≦large^56^. T, time effect; G, group effect.

**Table S2.** Correlation between body composition and sex-related hormone parameters.

| **Variables** | **Value** | Δ Testosterone | Δ Estradiol | Δ SHBG | Δ DHEA-S |
| --- | --- | --- | --- | --- | --- |
| Δ Weight | *r* | -.168 | .064 | -.374 | .213 |
|  | *p* | .433 | .767 | .072 | .317 |
| Δ BMI | *r* | -.157 | .067 | -.372 | .204 |
|  | *p* | .463 | .756 | .074 | .339 |
| Δ Waist circumference | *r* | -.556 | -.204 | -.352 | .020 |
|  | *p* | .005^**^ | .339 | .092 | .924 |
| Δ % abdominal fat | *r* | -.500 | -.288 | -.422 | -.220 |
|  | *p* | .013^*^ | .173 | .040^*^ | .302 |
| Δ Body fat mass | *r* | .534 | .216 | .334 | .379 |
|  | *p* | .007^**^ | .310 | .110 | .068 |
| Δ % body fat | *r* | -.534 | -.112 | -.436 | -.320 |
|  | *p* | .007^**^ | .603 | .033^*^ | .127 |
| Δ Skeletal muscle mass | *r* | .562 | .155 | .390 | .216 |
|  | *p* | .004^**^ | .471 | .060 | .310 |
| Δ Lean body mass | *r* | .534 | .311 | .248 | .411 |
|  | *p* | .010^*^ | .139 | .242 | .046^*^ |

Note: BMI=Body mass index. ^*^*p* < 0.05, ^**^*p* < 0.01.
